# Hyaluronic Acid Synthesis Contributes to Tissue Damage in Systemic Lupus Erythematosus

**DOI:** 10.3389/fimmu.2019.02172

**Published:** 2019-09-13

**Authors:** Abel Suarez-Fueyo, Maria G. Tsokos, Seung-Ki Kwok, Kayaho Maeda, Eri Katsuyama, Peter H. Lapchak, George C. Tsokos

**Affiliations:** Department of Medicine, Beth Israel Deaconess Medical Center, Harvard Medical School, Boston, MA, United States

**Keywords:** hyaluronic acid, systemic lupus erythematosus, extracellular matrix, 4-MU, autoimmunity

## Abstract

Hyaluronic acid (HA), a component of the extracellular matrix, is the ligand for CD44 and has been implicated in the pathogenesis of kidney inflammation in patients with systemic lupus erythematosus (SLE), but its direct role and mechanism of action have not been studied. Here we show that administration of hymecromone (4-Methylumbelliferone, 4-MU), an HA synthesis inhibitor, to lupus-prone mice suppressed dramatically lupus-related pathology. Interestingly, 4-MU stopped the appearance of disease when administered prior to its onset and inhibited the progression of disease when administered after its appearance. Inhibition of HA synthesis *in vivo* reduced tissue damage and the number of intrarenal lymphoid cell infiltrates including double negative CD3+CD4–CD8– T cells which are known to be involved in the pathogenesis of SLE. Exposure of human peripheral blood mononuclear cells to HA *in vitro* increased the generation of CD3+CD4–CD8– T cells through a mechanism involving Rho-associated kinase. Our results signify the importance of the HA-rich tissue microenvironment in the activation of lymphocytes to cause tissue damage in SLE and suggest the consideration of inhibition of HA synthesis to treat patients.

## Introduction

Systemic lupus erythematosus (SLE) is a chronic autoimmune disease affecting multiple organs. Skin and kidney inflammation and damage are frequent and lead to significant morbidity and mortality (1, 2).

The extracellular matrix (ECM), a three-dimensional macromolecular network composed of collagen, proteoglycans/glycosaminoglycans, elastin, fibronectin, laminins, and several other glycoproteins has been classically considered to provide structural support and to maintain the architecture of tissues and organs (3). Although this remains true, it is now clear that ECM components are important in the development of the inflammatory processes (4). Hyaluronic acid (HA) is a component of the ECM and has been claimed to be involved in the pathogenesis of autoimmune and malignant diseases (5). The synthesis of HA involves UDP-glucuronosyltransferase (UGT), which generates UDP-glucuronic acid (UDP-GlcUA) and UDP-N-acetyl-glucosamine (UDP-GlcNAc) by the transfer of an UDP-residue to N-acetylglucosamine and glucuronic acid, and HA synthases, which catalyze the transglycosylation from both UDP-GlcUA and UDP-GlcNAc (6). CD44 is a glycoprotein which binds HA and is important for the adhesion and migration of T cells (7).

Activation and expansion of pathogenic Th1, Th17, and TCRαβ+CD4–CD8– double-negative (DN) T cells are prominent in patients with SLE (8). We have previously shown that T cells from patients with SLE express increased amounts of CD44 which enables their binding to surfaces coated with HA (9) and CD3+CD44+ T cells are associated with increased disease activity (10).

After we confirmed that kidney and skin tissues from patients with SLE and lupus-prone mice express increased amounts of HA we considered that blockade of UGT should result in decreased HA expression and limited accumulation of immune cells to tissues. We report that treatment of MRL/*lpr* mice with hymecromone (4-Methylumbelliferone, 4-MU), a competitive inhibitor of UGT (6), reduces tissue inflammation and damage. Our studies further signify the importance of considering tissue-directed approaches (11) to suppress inflammation in SLE and probably in other autoimmune diseases.

## Materials and Methods

### Human Samples

Human kidney tissues were collected from three patients with lupus nephritis (26-year-old woman with class III nephritis, 41-year-old woman, with class IV-G nephritis and 25-year-old woman with class V nephritis) and from two patients with renal cell carcinoma from which healthy kidney areas were selected (45-year-old woman and 35-year-old woman) at the St. Mary's Hospital of the Catholic University of Korea. This study was approved by the Institutional Review Board of Seoul St. Mary's Hospital of the Catholic University of Korea (KC16SISI0980).

Healthy donors were recruited among staff at Beth Israel Medical Center. They provided informed consent approved by the Institutional Review Board. Peripheral blood from the study subjects was collected in lithium heparin tubes followed by density-gradient centrifugation (Lymphocyte Separation Medium; Corning Life Sciences). Double negative T (DN T) cells were expanded as previously reported (12). Briefly, peripheral blood mononuclear cells (PBMCs) were cultured for 5 days in RPMI 1640 with 10% FCS in 12-well plates which had been pre-coated with anti-CD3 antibody (5 μg/mL; BD Pharmingen) in the presence or absence of 100 μg/mL of hyaluronic acid sodium salt from Streptococcus equi (Sigma-Aldrich). In some experiments, fasudil [10 μM] was added for the duration of the experiment and renewed every other day.

### Mice

MRL/*lpr* mice were purchased from the Jackson Laboratory. All mice were maintained under specific pathogen-free conditions. Studies were performed using MRL/*lpr* male mice, which unlike the female mice develop disease at a slower pace and offer a wider therapeutic window to test the curative and interrupted treatment regiments. Female mice were used in confirmatory experiments. Urine protein levels were assessed with Multistix 10SG strips (SIEMENS) every 7 days.

### Treatment

A suspension of 4-MU (31 mg/mL, Sigma Aldrich) was prepared in MediDrop Sucralose (Clear H_2_O). Age- and sex-matched mice were exposed to *ad libitum* drinking of MediDrop Sucralose containing 4-MU (treated groups) or lacking 4-MU (control groups). We determined that MRL/*lpr* mice had an average MediDrop Sucralose intake of 8 mL/day. We calculated this based on the initial and final volume registered in two independent experiments with four mice in each one. Based on this measurement, the estimated dose is 250 mg/mouse/day. We did not observe a significant difference in the liquid intake between treated and control mice. Therefore, the observed differences should not be attributed to a possible effect of sucralose on the gut microbiome (13). All mice used for HA experiments were conditioned for 2 weeks with MediDrop Sucralose, starting at 3 months. Preventive treatment was initiated in 3.5 month-old mice and administered for 2 months, while curative treatment was initiated in 4.5 month-old mice and administered for 1 month. In a third group of mice, the treatment was initiated at 3.5 months of age and interrupted at 4.5 months of age (interrupted treatment). Mice were euthanized at 5.5 months of age, unless otherwise dictated by earlier development of severe proteinuria (vehicle treated mice), as stated in figure legends.

### Histopathologic Evaluation

Kidneys and skin were embedded in paraffin and sections were prepared and stained with hematoxylin and eosin (H&E), and periodic acid–Schiff (PAS) stains. Histological scoring of kidney samples was performed blindly by a nephropathologist according to a previously described scoring system (14). Skin lesions in the nose, ears, and interscapular region were examined macroscopically and scored using a scoring system from 0 to 3 [0 none, 1 mild, 2 moderate (<2 cm), 3 severe (≥ 2 cm)], as previously described (15). Histological scoring of skin sections from the interscapular region was performed blindly by a pathologist according to slightly modified previously described scoring systems (16, 17). Histologic parameters included epidermal hyperplasia (0 none, 1 mild, 2 severe), hyperkeratosis (0, none, 1 focal, 2 diffuse), ulcer (0 absent, 1 present), liquefaction of the basal layer (0 none, 1 focal, 2 diffuse), and dermal inflammatory infiltrates (0 none, 1 focal, 2 diffuse). For immunohistochemistry, sections were deparaffinized, treated with citrate buffer for antigen retrieval, blocked with an avidin/biotin blocking kit (Vector), and stained with biotinylated HA binding protein (HABP, Amsbio), followed by washing in phosphate buffered saline (PBS) and incubation with streptavidin-horseradish peroxidase (HRP) reagent (Vector). Signal development was performed using 3,3′-diaminobenzidine (DAB, Vector). Quantification of HA expression was performed by ImageJ color deconvolution using hematoxylin and DAB (H-DAB) vector on several kidney images covering the entire area of the kidney.

### Flow Cytometry

Spleen and lymph nodes were excised from mice, and single-cell suspensions were obtained by teasing the organs through a nylon mesh. Perfused kidneys with PBS were digested with collagenase type 4 (100 μg/mL) (Worthington Biochemical) in HBSS for 30 min (37°C) to isolate single cells. Isolated cells were stained for flow cytometry with antibodies against B220 (clone RA-3-6B2, BioLegend), CD3 (clone 145-2C11, BioLegend), CD4 (clone GK1.5, BioLegend), CD8a (clone 53-6.7, BioLegend), CD25 (clone PC61, BioLegend), CD44 (clone IM7, BioLegend), CD45 (clone 30-F11, BioLegend), CD62L (clone MEL-14, BioLegend), CD138 (clone 281-2, Biolegend), CD185 (clone L138D7, BioLegend), CD279 (clone RMP1-30, BioLegend) and TCRβ (clone H57-597, BioLegend) for 30 min at 4°C. Total cell numbers were determined using beads (Spherotech) and counting live cells based on forward scatter and side scatter. Absolute cell numbers were calculated on the basis of the percentage of each population. For intracellular cytokine staining, cells were isolated as described above and stimulated for 4 h in culture medium containing PMA (5 ng/mL; Sigma-Aldrich), ionomycin (500 ng/mL; Sigma-Aldrich), and brefeldin A (GolgiStop; BD Biosciences). After staining of surface markers for 30 min at 4°C, cells were fixed and permeabilized with Cytofix/Cytoperm and Perm/Wash buffer according to the manufacturer's instructions (BD Biosciences). Subsequently, cells were stained with antibodies to anti–IFNγ (clone XMG1.2, BioLegend). For FOXP3 staining, cells were permeabilized using the Mouse Regulatory T cell Staining Kit (eBioscience) and anti-FOXP3 antibody (clone MF-14, eBioscience). Human PBMCs were stained following the same protocol with antibodies against CD3 (clone UCHT1, BioLegend), CD4 (clone RPA-T4, BioLegend), CD8 (clone RPA-T8, BioLegend), IFNγ (clone 4S.B3, BioLegend), and TCRαβ (clone IP26, BioLegend). All flow cytometry data were acquired on a Gallios or Cytoflex (Beckman Coulter) and analyzed with Kaluza software (Beckman Coulter).

### Statistics

All results are shown as the mean ± SEM. GraphPad Prism V6.0 software was used for statistical analysis. The applied statistical tests are indicated in each figure legend. *P*-values of <0.05 were considered significant. The data were presented as one of several independent experiments. When sufficiently large numbers of mice were required for statistical analysis we combined numbers of mice from separate experiments conducted using identical protocols.

## Results

### HA Is Highly Expressed in the Kidneys of Patients With SLE and MRL*/lpr* Mice

**Figure 1:**
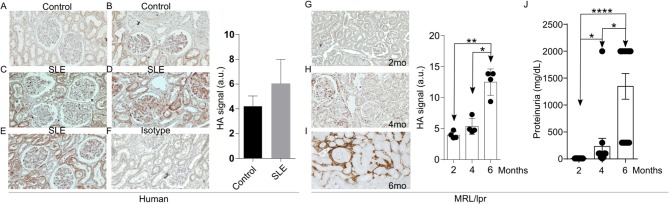
Increased HA expression in the kidneys of patients with SLE and lupus-prone MRL/*lpr* mice. **(A–F)** Representative pictures of immunohistochemistry staining for HA (Original magnification 20X) and quantitative cumulative data (a.u., arbitrary units) showing expression of HA in human kidney tissues. **(A)** Normal kidney; **(B)** Normal kidney; **(C)** SLE nephritis class III; **(D)** SLE nephritis class IV-G; **(E)** SLE nephritis class V; **(F)** Negative control (SLE nephritis class IV-G stained only with streptavidin-HRP); **(G–I)** Representative pictures of immunohistochemical staining for HA (Original magnification 10X) and quantitative cumulative data of HA expression in kidney tissues from male MRL/*lpr* mice at ages 2–6 months (*n* = 4 mice in each group). Paired ANOVA analysis with multiple comparisons ^*^p < 0.05, ^**^p < 0.01. **(G)** 2 months; **(H)** 4 months; **(I)** 6 months; **(J)** Cumulative data showing proteinuria in MRL/*lpr* mice at ages 2–6 months (*n* = 13 mice). Paired ANOVA analysis with multiple comparisons. ^*^p < 0.05, ^****^p < 0.0001.

Increased expression of the V3 and V6 isoforms of CD44 on T cells from patients with SLE was found to correlate with disease activity (10). More interestingly, silencing of CD44 in T cells from patients with SLE deprives them of their ability to adhere to membranes coated with HA through a ROCK-dependent mechanism and inhibits their migration into tissues (9). HA is a ligand for CD44 and its levels have been reported increased in the sera and kidneys of patients and mice with active lupus nephritis (18, 19). This information prompted us to ask whether HA is involved in the recruitment of T cells into tissues to promote inflammation. We first confirmed the expression and distribution of HA in kidney sections from three patients with SLE nephritis and in normal kidney tissue from two renal carcinoma patients of same-sex and similar age by immunohistochemistry (Figures 1A–E). While the normal kidney tissue expressed minimal amounts of HA (Figures 1A,B), tissues from patients with lupus nephritis displayed significantly higher HA expression within glomeruli and tubular epithelial cells (Figures 1C–E) and were entirely negative when stained with streptavidin-HRP in the absence of HA binding protein (negative control, Figure 1F). Next, we evaluated the expression of HA in the kidneys of MRL/*lpr* lupus-prone mice. Similar to human tissues, HA expression had a glomerular, periglomerular, and tubulointerstitial distribution, increased progressively as the mice aged and the disease worsened, being localized predominantly in periglomerular and peritubular areas in the aged mice (Figures 1G–I), and correlated with the development of proteinuria (Figure 1J). Prevalent expression in periglomerular instead of intraglomerular areas in the older lupus-prone mice may be explained by *in vitro* data showing that most of the newly synthesized HA by renal mesangial cells exposed to antibodies from lupus patients is secreted in the conditioned medium (16).

### Inhibition of HA Synthesis Prior to the Onset of Disease Reduced Lymphocyte Infiltration and Prevented Tissue Damage in MRL/*lpr* Mice

**Figure 2:**
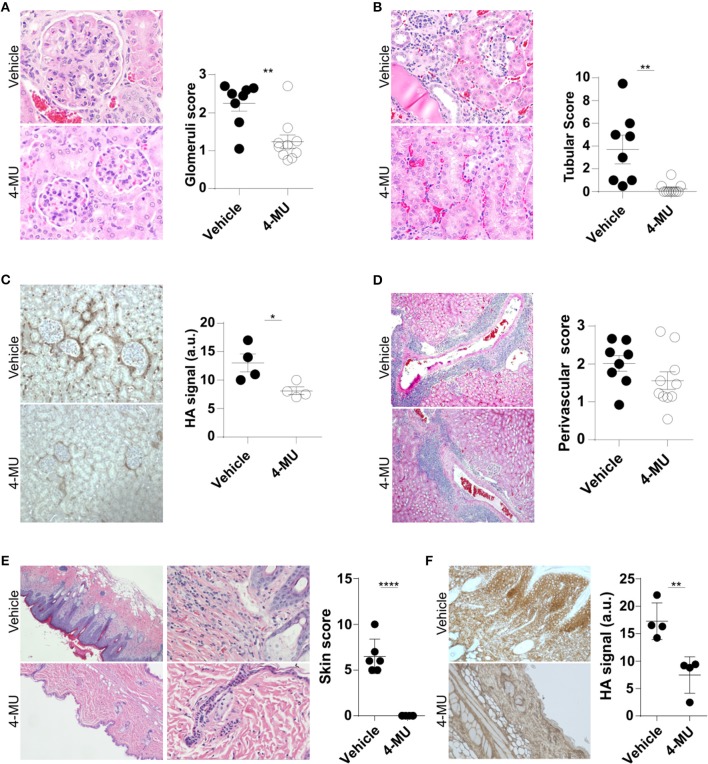
Treatment with 4-MU prior to development of disease decreases expression of HA and prevents tissue damage in MRL*/lpr* mice. **(A)** Representative pictures of glomerular histologic changes and cumulative data showing the pathologic scoring of glomeruli in kidneys from male mice treated with vehicle or 4-MU. H&E, original magnification 40X. Student's *t*-test. ^**^p < 0.01. **(B)** Representative pictures of tubular histologic changes and cumulative data showing the pathologic scoring of tubules in kidneys from male mice treated with vehicle or 4-MU. H&E, original magnification 40X. Student's *t*-test. ^**^p < 0.01. **(C)** Representative pictures and quantitative cumulative data showing expression of HA in kidneys from male mice treated with vehicle or 4-MU. Original magnification 20X. Student's *t*-test. ^*^p < 0.05. **(D)** Representative pictures of histologic changes and cumulative data showing the pathologic scoring of perivascular areas in kidneys from male mice treated with vehicle or 4-MU. H&E, original magnification 10X. Student's *t*-test. **(E)** Representative pictures showing histologic changes and cumulative data showing the histopathologic scoring in the skin of male mice treated with vehicle or 4-MU. H&E, original magnifications 4X (left) and 20X (right). Student's *t*-test. ^****^p < 0.0001. **(F)** Representative pictures and quantitative cumulative data of HA expression in the skin of mice treated with vehicle or 4-MU. Original magnification 4X. Student's *t*-test. ^**^p < 0.01. **(A,B,D)** *n* = 8 mice in vehicle and *n* = 10 mice in 4-MU treated groups from four independent experiments. **(C)** *n* = 4 mice in each group from a representative experiment out of four independent experiments. **(E)** *n* = 6 mice in each group from two independent experiments. **(F)** *n* = 4 mice in each group. Tissues were collected from mice sacrificed at 5.5. months of age.

Our finding that the expression of HA in the kidney of lupus-prone mice increases in parallel with their age and levels of proteinuria suggested that HA is involved in the pathogenesis of the disease. To address in a definitive manner the link between HA expression and lupus pathology, we evaluated histologically the kidneys of MRL/*lpr* mice before and after treatment with the inhibitor of HA synthesis hymecromone (4-Methylumbelliferone, 4-MU) (20). By administering the inhibitor prior to the appearance of clinical disease (3.5 month old mice), we sought to determine whether inhibition of HA synthesis can prevent disease development. We found that the 4-MU-treated mice had a significant reduction in the number of intrarenal lymphocytic infiltrates (Supplemental Figure 1) and in the magnitude of glomerular and tubular damage (Figures 2A,B), along with reduced expression of HA in the same areas of the kidneys (Figure 2C), when compared to the vehicle-treated mice. We did not observe significant differences in the infiltration of lymphocytes in the perivascular areas (Figure 2D).

Additionally, we found that treated mice did not develop skin disease. Histologic examination of the skin showed that the 4-MU-treated MRL/*lpr* mice lacked acanthosis, hyperkeratosis and intradermal lymphocytic cell infiltrates and retained a thin epidermis (Figure 2E), that was negative for HA, in contrast to the vehicle-treated mice which exhibited a hyperplastic epidermis with strong diffuse expression of HA (Figure 2F).

### Inhibition of HA Synthesis After the Onset of Disease Halted Its Progression in MRL*/lpr* Mice

**Figure 3:**
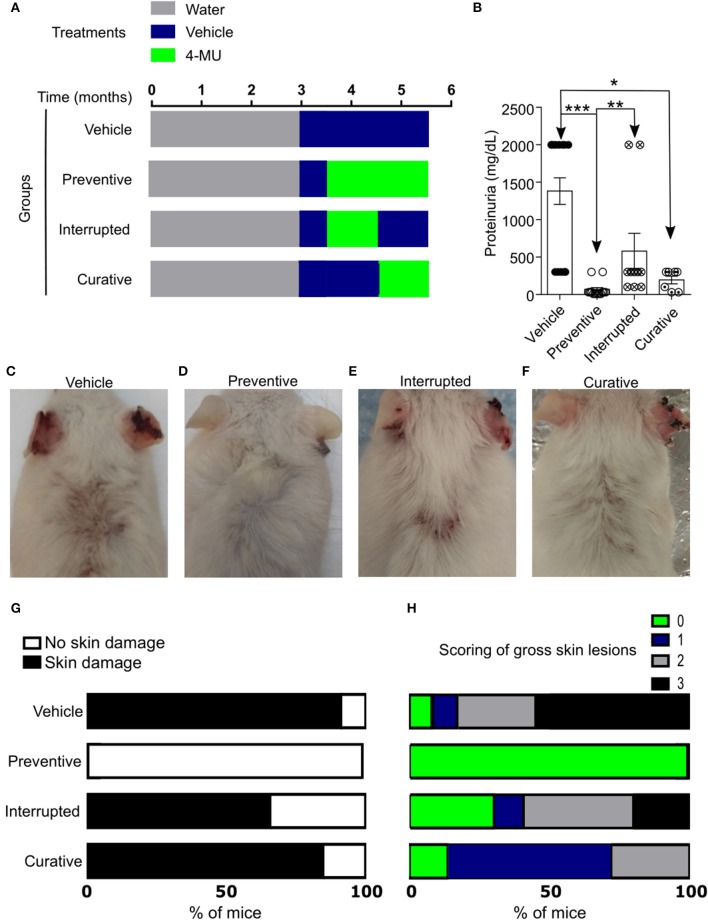
Inhibition of HA synthesis prevents the development or progression of disease in MRL*/lpr* mice. **(A)** Scheme showing start- and end-points of the treatment schemes. **(B)** Cumulative data showing proteinuria (mg/dL) in male mice at 5.5 (preventive, interrupted, curative treatment) and 4.5–5.5 (vehicle treatment) months of age. Vehicle, *n* = 13; Preventive treatment, *n* = 14; Interrupted treatment, *n* = 10; Curative treatment, *n* = 7 mice from 2 to 4 independent experiments. ANOVA, multiple comparisons with Kruskal-Wallis test. ^*^p < 0.05, ^**^p < 0.01, ^***^ *p* < 0.001. **(C–F)** Representative pictures showing gross skin damage in male mice treated with: **(C)** Vehicle; **(D)** 4-MU starting at 3.5 months of age (Preventive treatment); **(E)** 4-MU starting at the 3.5 months of age and interrupted at the 4.5 months of age (Interrupted treatment); **(F)** 4-MU starting at the 4.5 months of age (Curative treatment); **(G)** Frequency of skin involvement (disease penetrance) in the various treatment groups; **(H)** Percentage of mice with specific score of skin injury in the treatment groups shown in **(G)**. All skin images were obtained from 5.5 month old mice. Numbers, sex and age of mice used to calculate frequency and scoring of skin lesions are the same as in **(B)**.

In order to further understand the role of HA in the progression of the disease and its possible exploitation as a therapeutic target, we designed additional treatment schemes, which are summarized in Figure 3A and Supplemental Figure 2A consist of: (1) the preventive treatment group in which 4-MU was administered prior to the onset of clinical disease beginning at 3.5 months of age (2) the curative treatment group in which 4-MU was administered after the onset of clinical disease beginning at 4.5 months of age and (3) the interrupted treatment group in which 4-MU administration started prior to the appearance of clinical disease (at 3.5 months of age) and was interrupted at 4.5 months of age. All treatments were terminated at 5.5 months of age. In accordance with the data on kidney damage described above for the preventive treatment group, proteinuria did not develop when mice were treated with 4-MU starting at 3.5 months of age (Figure 3B, Supplemental Figure 2B), but appeared as soon as treatment was discontinued. When the treatment was initiated at 4.5 months of age, the existing proteinuria was not reversed, but did not progress as in the vehicle-treated mice (Figure 3B).

Similarly, gross evaluation of the skin showed that mice treated with 4-MU prior to the clinical onset of the disease did not develop skin lesions compared with those treated with vehicle alone (Figures 3C,D,G,H, Supplemental Figures 2C–F), whereas mice receiving interrupted treatment developed skin lesions almost immediately after the treatment was discontinued (Figures 3E,G,H). Also, skin lesions existing prior to the treatment with 4-MU at 4.5 months of age did not disappear or decrease in size, but did not progress as in the vehicle-treated mice (Figures 3F–H). These data suggest that the synthesis of HA contributes to the development of skin and kidney damage in lupus-prone mice and its inhibition controls disease progression.

### Inhibition of HA Synthesis in MRL*lpr* Mice Reduced Spleen Size and Cell Numbers

**Figure 4:**
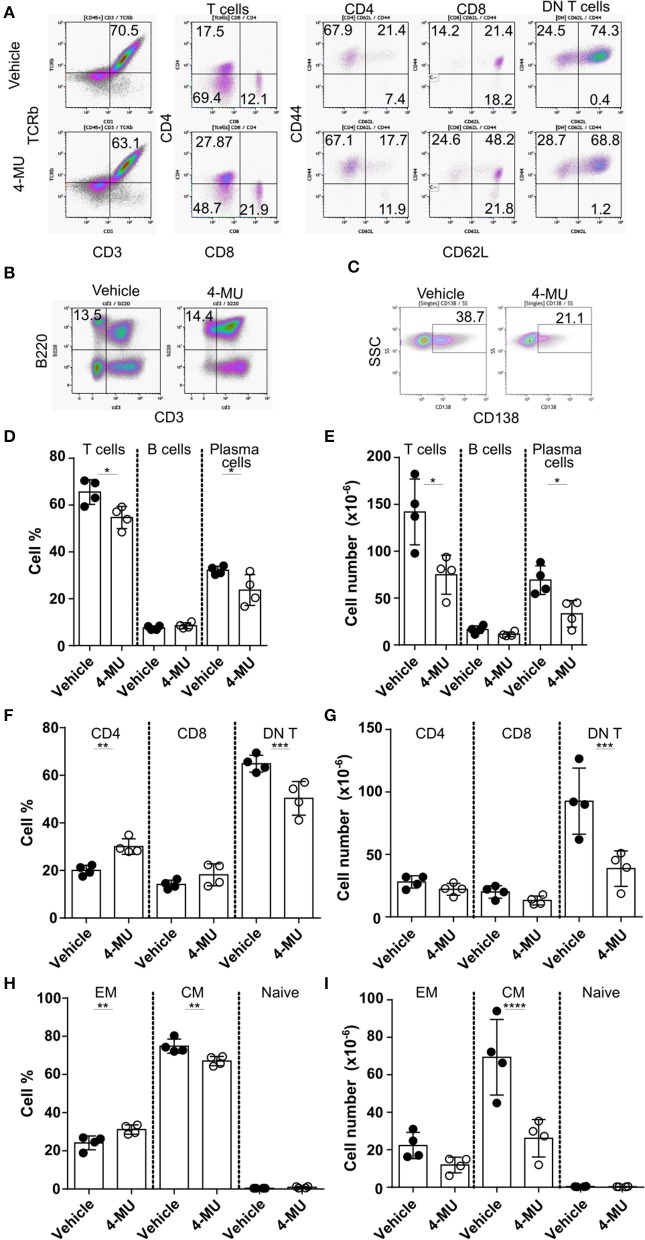
Preventive treatment with 4-MU treatment reduces the numbers of DN T and plasma cells in MRL/*lpr* mice. **(A–C)** Density plots showing the gating strategy for **(A)** T cell subpopulations, **(B)** B cells, and **(C)** plasma cells. **(D)** Cumulative data showing percentage of T cells (TCRβ+CD3+), B cells (B220+CD3–) and plasma cells (CD138+) in the spleen of male mice treated with vehicle or 4-MU. Student's *t*-test comparing both treatment groups for each type of cell. ^*^p < 0.05. **(E)** Cumulative data showing number of T cells (TCRβ+CD3+), B cells (B220+CD3–), and plasma cells (CD138+) in the spleen of male mice treated with vehicle or 4-MU. Student's *t*-test comparing both treatment groups for each type of cell. ^*^p < 0.05. **(F)** Cumulative data showing percentage of CD4, CD8, and DN T cells in the spleen of male mice treated with vehicle or 4-MU. Student's *t*-test comparing both treatment groups for each type of cell. ^**^p < 0.01, ^***^p < 0.001. **(G)** Cumulative data showing number of CD4, CD8, and DN T cells in the spleen of male mice treated with vehicle or 4-MU. Student's *t*-test comparing both treatment groups for each type of cell. ^***^p < 0.001. **(H)** Cumulative data showing percentage of effector memory (EM, CD44+CD62L–), central memory (CD44+CD62L+), and naive (CD44–CD62L+) DN T cells in the spleen of male mice treated with vehicle or 4-MU. Student's *t*-test comparing both treatment groups for each type of cell. ^**^p < 0.01. **(I)** Cumulative data showing number of effector memory (EM, CD44+CD62L–), central memory (CD44+CD62L+), and naive (CD44–CD62L+) DN T cells in the spleen of male mice treated with vehicle or 4-MU. Student's *t*-test comparing both treatment groups per each type of cell. ^****^p < 0.0001. Data from a representative experiment out of four independent experiments. Cells were collected from mice sacrificed at 5.5 months of age.

Treatment of MRL*lpr* mice with 4-MU resulted in decreased spleen size and cell numbers compared to animals treated with the vehicle alone (Supplemental Figures 3A,B). We analyzed by flow cytometry the cellular populations to determine whether inhibition of HA synthesis affects specific cell populations. We noted that inhibition of HA synthesis affected mainly the T cell compartment (CD3+TCRβ+) and plasma cells (CD138+) (Figures 4A–E). Analysis of T cell populations showed that inhibition of HA synthesis affected primarily the numbers of DN (CD3+CD4-CD8) T cells (Figures 4F,G) and especially those with a central memory phenotype which are characterized by high expression of CD44 (Figures 4H,I).

### 4-MU Suppressed the Pro-Inflammatory Profile of T Cells

**Figure 5:**
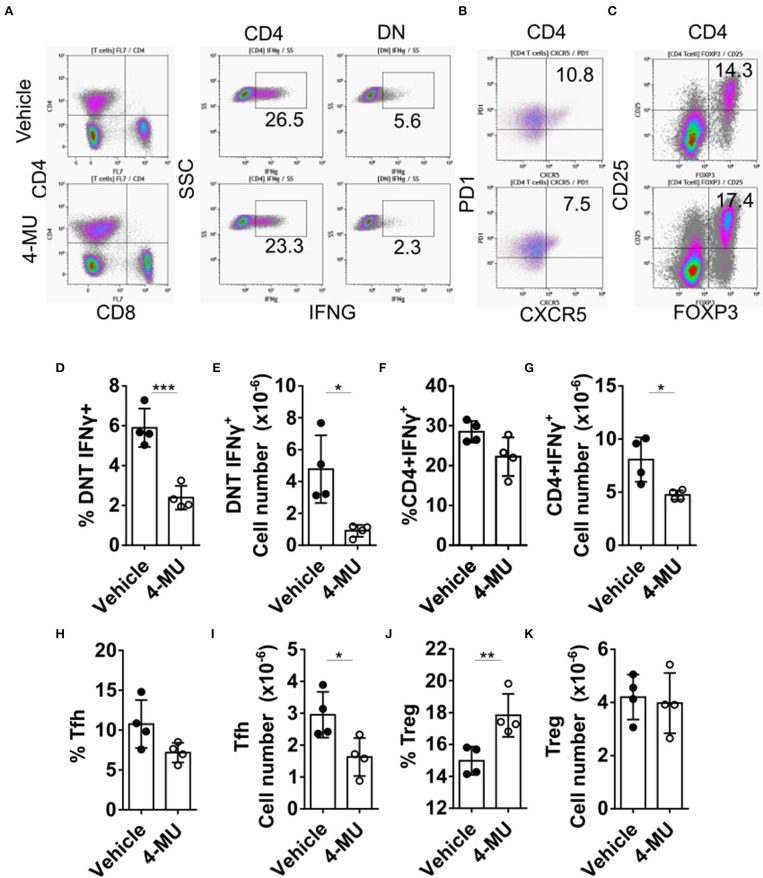
Preventive treatment with 4-MU mitigates the pro-inflammatory profile of T cells. **(A–C)** Density plots showing the gating strategy for **(A)** IFNγ producing cells, **(B)** Tfh cells, and **(C)** regulatory T cells. **(D)** Cumulative data showing percentage of DN T cells producing IFNγ in the spleens of male mice treated with vehicle or 4-MU. Student's *t*-test. ^***^p < 0.001. **(E)** Cumulative data showing number of DN T cells producing IFNγ in the spleens of male mice treated with vehicle or 4-MU. Student's *t*-test. ^*^p < 0.05. **(F)** Cumulative data showing percentage of CD4 cells producing IFNγ in the spleens of male mice treated with vehicle or 4-MU. Student's *t*-test. **(G)** Cumulative data showing number of CD4 cells producing IFNγ in the spleens of male mice treated with vehicle or 4-MU. Student's *t*-test. ^*^p < 0.05. **(H)** Cumulative data showing percentage of Tfh cells (CD3+TCRβ+CD4+CXCR5+PD-1+) in the spleens of male mice treated with vehicle or 4-MU. Student's *t*-test. **(I)** Cumulative data showing number of Tfh cells (CD3+TCRβ+CD4+CXCR5+PD-1+) in the spleens of male mice treated with vehicle or 4-MU. Student's *t*-test. ^*^p < 0.05. **(J)** Cumulative data showing percentage of Treg cells (CD3+TCRβ+CD4+CD25+FOXP3+) in the spleens of male mice treated with vehicle or 4-MU. Student's *t*-test. ^**^p < 0.01. **(K)** Cumulative data showing number of Treg cells (CD3+TCRβ+CD4+CD25+FOXP3+) in the spleens of male mice treated with vehicle or 4-MU. Student's *t*-test. Data from a representative experiment out of four independent experiments. Cells were collected from mice sacrificed at 5.5 months of age.

Double-negative (DN) T cells are defined by the absence of CD4 and CD8 and the ability to produce proinflammatory cytokines like IFNγ which has been linked to SLE pathogenesis both in humans and mice (12). Administration of 4-MU decreased the proinflammatory phenotype of DN and CD4+ T cells as manifested by a statistically significant decrease in the percentage and number of cells able to produce IFNγ (Figures 5A–G), an essential cytokine for disease development in MRL/*lpr* mice (21). In addition, treatment with 4-MU led to a reduction of T follicular helper cells (CD3+TCRβ+CD4+CXCR5+PD1+, Figures 5H,I), which could account for the decreased numbers of plasma cells. The percentage of regulatory T (Treg) cells (Figure 5J) was increased in the treated mice although the total numbers remained the same (Figure 5K).

### 4-MU Reduced the Infiltration of T Cells in the Kidney of MRL/l*pr* Mice

**Figure 6:**
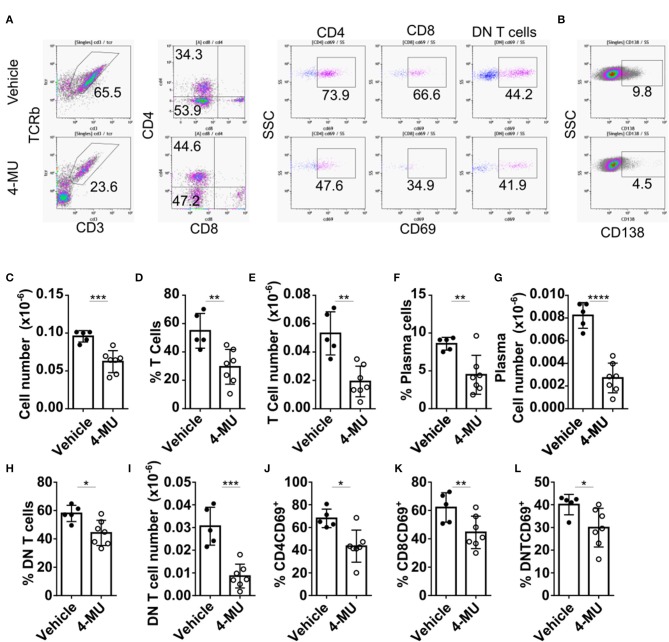
Preventive treatment with 4-MU decreases kidney cell infiltration and T cell activation. **(A,B)** Density plots showing the gating strategy for **(A)** T cell subpopulations and **(B)** plasma cells. **(C)** Cumulative data showing the total numbers of infiltrating lymphocytes (CD45+) in the kidneys of male mice treated with vehicle or 4-MU. Student's *t*-test. ^***^p < 0.001. **(D)** Cumulative data showing the percentage of infiltrating T cells in the kidneys of male mice treated with vehicle or 4-MU. Student's *t*-test. ^**^p < 0.01. **(E)** Cumulative data showing the total number of infiltrating T cells in the kidneys of male mice treated with vehicle or 4-MU. Student's *t*-test. ^**^p < 0.01. **(F)** Cumulative data showing the percentage of infiltrating plasma cells in the kidneys of male mice treated with vehicle or 4-MU. Student's *t*-test. ^**^p < 0.01. **(G)** Cumulative data showing the total number of infiltrating plasma cells in the kidneys of male mice treated with vehicle or 4-MU. Student's *t*-test. ^****^p < 0.0001. **(H)** Cumulative data showing the percentage of infiltrating DN T cells in the kidneys of male mice treated with vehicle or 4-MU. Student's *t*-test. ^*^p < 0.05. **(I)** Cumulative data showing the total number of infiltrating DN T cells in the kidneys of male mice treated with vehicle or 4-MU. Student's *t*-test. ^***^p < 0.001. **(J)** Cumulative data showing the percentage of infiltrating CD4 T cells expressing CD69 in the kidneys of male mice treated with vehicle or 4-MU. Student's *t*-test. ^*^p < 0.05. **(K)** Cumulative data showing the percentage of infiltrating CD8 T cells expressing CD69 in the kidneys of male mice treated with vehicle or 4-MU. Student's *t*-test. ^**^p < 0.01. **(L)** Cumulative data showing the percentage of infiltrating DN T cells expressing CD69 in the kidneys of male mice treated with vehicle or 4-MU. Student's *t*-test. ^*^p < 0.05. Vehicle *n* = 5 and 4-MU *n* = 7 mice from two experiments. Cells were collected from mice sacrificed at 5.5 months of age.

The reduction of cellular infiltrates in the kidney of 4-MU-treated mice that was initially observed by histologic examination (Supplemental Figure 1), was quantified by flow cytometry and found to be statistically significant (Figure 6C). Flow cytometric analysis further showed reduced percentages and numbers of T cells (Figures 6A–D) and plasma cells (Figures 6F,G) in the kidneys of mice treated with 4-MU compared to those treated with vehicle alone. As in the spleen, the numbers of the DN T cells were the most affected T cell subpopulation in the kidney (Figures 6H,I). CD4 and CD8 T cell numbers were also reduced in the treated mice (Supplemental Figures 4A–D). Furthermore, the expression of the activation marker CD69 was decreased on all T cell subsets, i.e., CD4 (Figure 6J), CD8 (Figure 6K), and DN T cells (Figure 6L). These findings demonstrate that high HA expression promotes the infiltration of lymphocytes, plasma cells, activated T cells and proinflammatory DN T cells into the kidney.

### HA Contributes to the Expansion of DN T Cells in Humans

**Figure 7:**
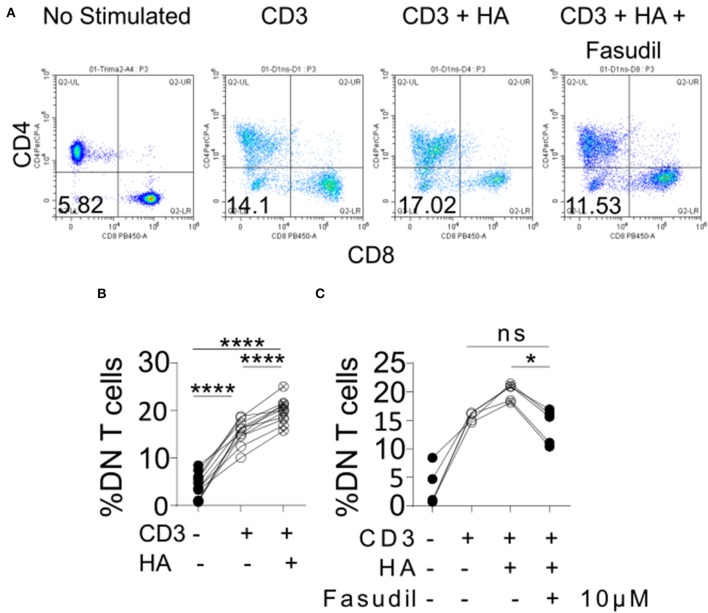
Exposure of human peripheral blood mononuclear cells to HA expands DN T cells. **(A)** Density plots showing the gating strategy for DN T cells. **(B)** Cumulative data showing percentage of DN T cells (gated as TCRab+CD3+CD4–CD8–) after *in vitro* stimulation (*n* = 11). Paired ANOVA with Greenhouse-Geisser correction and multiple comparisons Turkey's correction. ^****^p < 0.0001. **(C)** Cumulative data showing the effect of 10 μM of fasudil in the expansion of DN T cells (*n* = 5, gated as TCRab+CD3+CD4–CD8–) Paired ANOVA with Greenhouse-Geisser correction and multiple comparisons Turkey's correction. ^*^p < 0.05.

Among the kidney-infiltrating T cells the DN subset was reduced most prominently in mice treated with 4-MU. This observation prompted us to ask whether the increased expression of HA in the kidney contributed to the expansion of this proinflammatory subset in humans. To this end, we exposed peripheral blood mononuclear cells from healthy donors to HA i*n vitro*. Indeed, the number of generated DN T cells after stimulation with a CD3 antibody (12) increased significantly in the presence of HA compared to cells stimulated in the absence of HA (Figures 7A,B). Because HA is a ligand for CD44 and we had previously shown that T cells with high CD44 expression infiltrate the kidney through activation of Rho-associated kinase (ROCK), which is important in securing their adhesive capacity (9), we asked whether ROCK is involved in the HA-induced expansion of DN T cells. Indeed, addition in the cultures of the ROCK inhibitor fasudil limited significantly the HA-induced expansion of DN T cells (Figure 7C), revealing the importance of the HA-CD44-mediated signaling in the expansion of the pathogenic DN T cells in patients and mice with SLE.

## Discussion

An important role has been recognized for HA, a component of the ECM, in the development of autoimmune disorders such as diabetes (22), multiple sclerosis (23–25) and rheumatoid arthritis (26). In this communication, we present evidence on the importance of HA synthesis and expression in the development of the inflammatory response in tissues of lupus-prone mice. After we confirmed that HA expression is increased in the kidneys of patients with lupus nephritis and lupus-prone MRL/*lpr* mice, we demonstrated that inhibition of its synthesis mitigates tissue inflammation by decreasing the invasion of proinflammatory DN T cells. Interestingly, we observed that the therapeutic effect depends on the continuous presence of the drug/inhibitor of HA synthesis.

Previous reports had indicated that HA expression is increased in the kidneys of patients with SLE and MRL/*lpr* mice and levels of circulating HA are increased in the sera of patients with SLE (18, 19), but a pathogenic role had not been established. Here we show that HA which we found to be increased not only in the kidney but also in the skin of MRL/*lpr* mice is involved directly in the induction of kidney and skin inflammation and tissue damage because the commercially available inhibitor of HA synthesis 4-MU mitigated disease. The fact that 4-MU has been approved to treat patients with biliary dyskinesia (27) in several countries provided additional impetus for our studies. Indeed, treatment with 4-MU limited the expression of HA in the tissues and this was associated with clinical improvement and limited the presence of the proinflammatory DN T cells.

Previously we had shown that CD44 is expressed in increased amounts on the surface of T cells from patients with SLE (10) and had found the levels of expression to correlate with disease activity (10). In addition CD44 positive cells were found in the kidneys of patients with SLE (9, 12). More importantly, we reported that silencing of CD44 suppresses the ability of T cells to adhere to membranes coated with HA and inhibits their migration into tissues (9). Here we demonstrate the significance of HA, which is synthesized in excess in the tissues of lupus-prone mice, in the expression of skin and kidney inflammation.

HA has been shown to contribute to the appearance of a proinflammatory profile in T cells (24), a finding which has been expanded with our data demonstrating that inhibition of HA synthesis reduces the numbers of IFNγ-producing and CD69- expressing DN T cells. Proinflammatory DN T cells are generated from CD8+ cells upon the encounter of an autoantigen (28). Here we show the contribution of the engagement of HA and the importance of ROCK in the propagation of DN T cells with proinflammatory features. The ROCK inhibitor fasudil has been reported to suppress skin lesions in patients with psoriasis vulgaris (29) and to limit the entry of T cells in the skin. Our finding that the HA synthesis inhibitor 4-MU accomplishes similar effects in mice suggests the possible beneficial effect of combined treatment with both drugs in lower doses to suppress tissue inflammation. Similar mechanisms may be involved in the infiltration of T cells in other tissues such as synovium (30) and prompt the design of additional therapeutic approaches (31).

In conclusion, our results highlight the importance of the ECM as well as the receptor for HA, CD44, and its signaling partner ROCK in the pathogenesis of SLE. In addition our study has substantial translational value since 4-MU is already approved to be used in patients in several countries.

## Data Availability

All datasets generated for this study are included in the manuscript and/or the Supplementary Files.

## Ethics Statement

This study was approved by the Institutional Review Board of Seoul St. Mary's Hospital of the Catholic University of Korea (KC16SISI0980) and by the Institutional Review Board of Beth Israel Deaconess Medical Center.

## Author Contributions

GT: conceptualization, funding acquisition, and supervision. AS-F, MT, S-KK, KM, EK, PL, and GT: methodology. AS-F, S-KK, and KM: investigation. AS-F, MT, S-KK, KM, EK, and GT: formal analysis. AS-F and GT: writing–original draft. AS-F, MT, and GT: writing–review and editing.

### Conflict of Interest Statement

The authors declare that the research was conducted in the absence of any commercial or financial relationships that could be construed as a potential conflict of interest.
