# Supplementary figures and images for: Hyaluronic Acid Synthesis Contributes to Tissue Damage in Systemic Lupus Erythematosus
Source: Front Immunol. 2019 Sep 13;10:2172. doi: 10.3389/fimmu.2019.02172 (PMC6753633; doi:10.3389/fimmu.2019.02172)

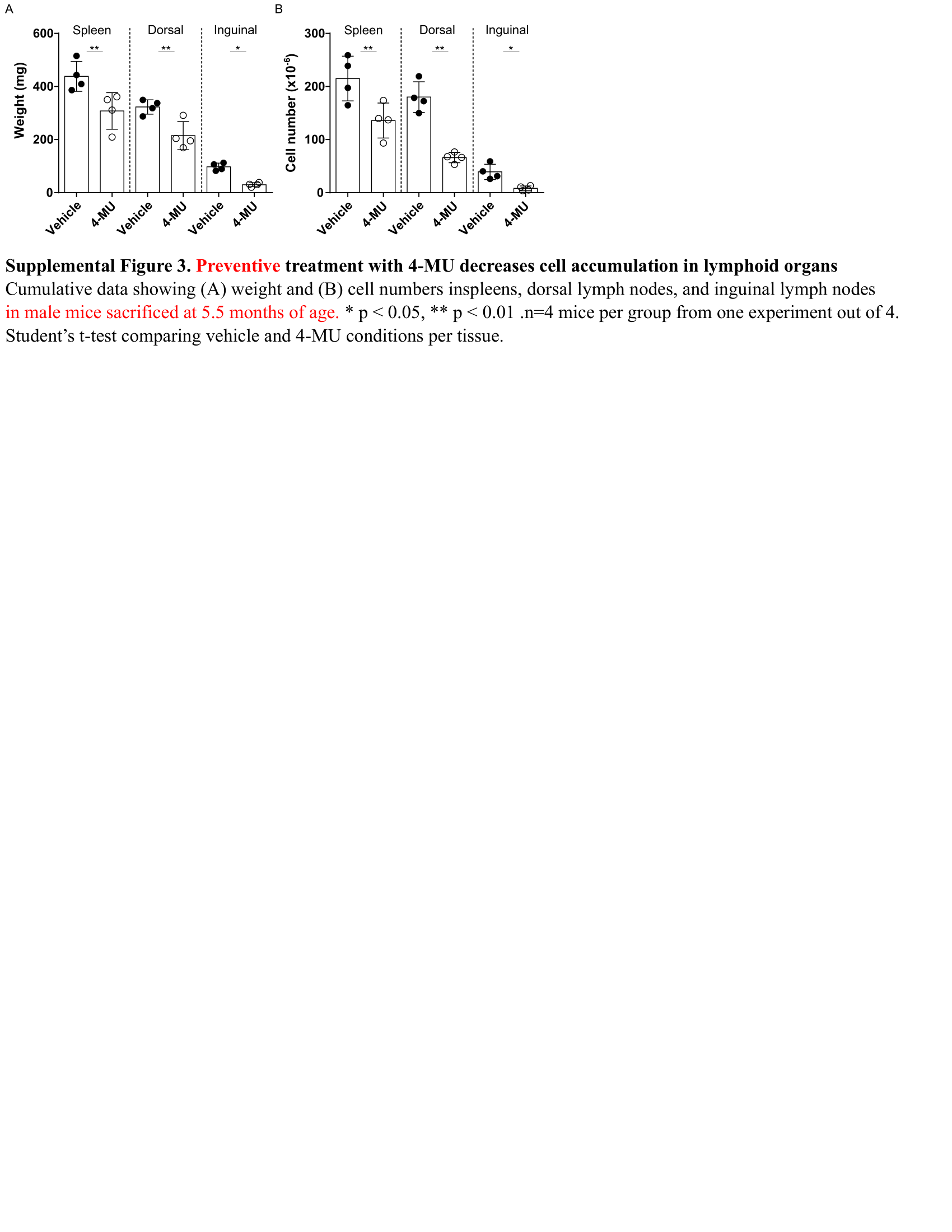

Supplement: Supplementary file 3 [file Image_3.TIFF]

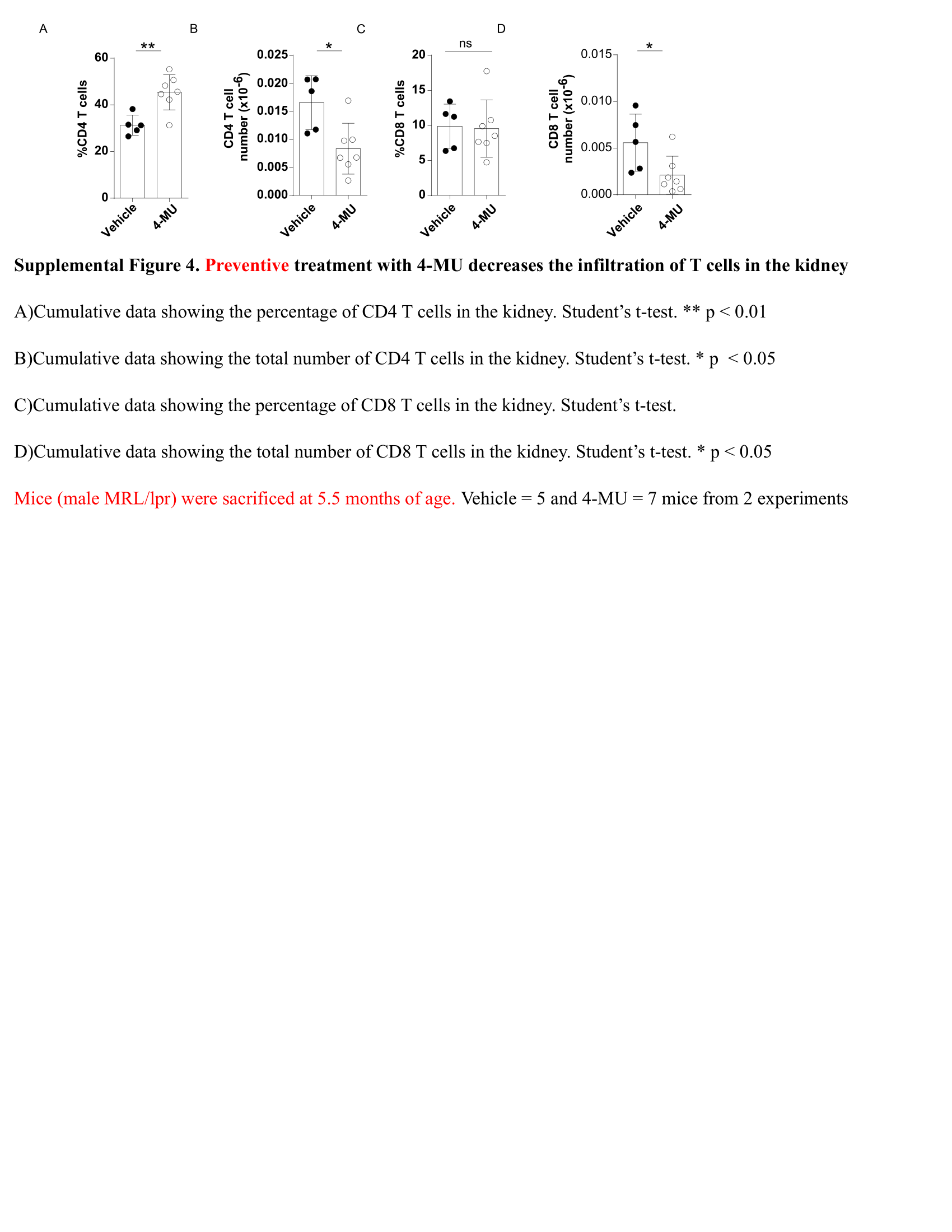

Supplement: Supplementary file 4 [file Image_4.TIFF]
